# Piezo-herbal microneedle patches enable wireless endometrial regeneration and fertility recovery

**DOI:** 10.1186/s12951-026-04313-5

**Published:** 2026-03-26

**Authors:** Rui Zhao, Shiwen Ni, Mei Yang, Zhifeng Gu, Yujuan Zhu

**Affiliations:** Research Center of Clinical Medicine, Affiliated Hospital of Nantong University, Medical School of Nantong University, Nantong, 226001 China

**Keywords:** Herbal hydrogel microneedle, Curcumin, Potassium-sodium niobate, Ultrasonic response, Endometrial repair

## Abstract

Endometrial injury is a major cause of female infertility and recurrent miscarriage. Conventional treatments often fail to dynamically regulate the local immune microenvironment or effectively promote angiogenesis and functional regeneration. Here, we developed an ultrasound-responsive microneedle (MN) patch for wireless endometrial repair, integrating the natural compound curcumin and piezoelectric potassium sodium niobate (KNN) nanoparticles into a photopolymerized hydrogel. This design enables spatiotemporally controlled drug release, antioxidant activity, and piezoelectric stimulation under ultrasound activation. In vitro, the Cur@KNN MN exhibited excellent biocompatibility, potent reactive oxygen species scavenging, anti-inflammatory effects, and pro-angiogenic capacity, as validated in macrophage and vascular-on-a-chip models. In a rat endometrial injury model, implantation of the patch combined with ultrasound exposure promoted KNN-mediated microcurrent generation, which synergistically enhanced curcumin release and significantly improved regeneration of smooth muscle and microvessels. Notably, the treated endometrium showed increased thickness, reduced fibrosis, and restored fertility, with a marked increase in embryo implantation rates. These results demonstrate that the traditional Chinese medicine-based piezoelectric MN system not only supports structural reconstruction of the injured uterus but also effectively restores reproductive function, offering an innovative and minimally invasive strategy for in situ tissue regeneration.

**Graphical Abstract:**

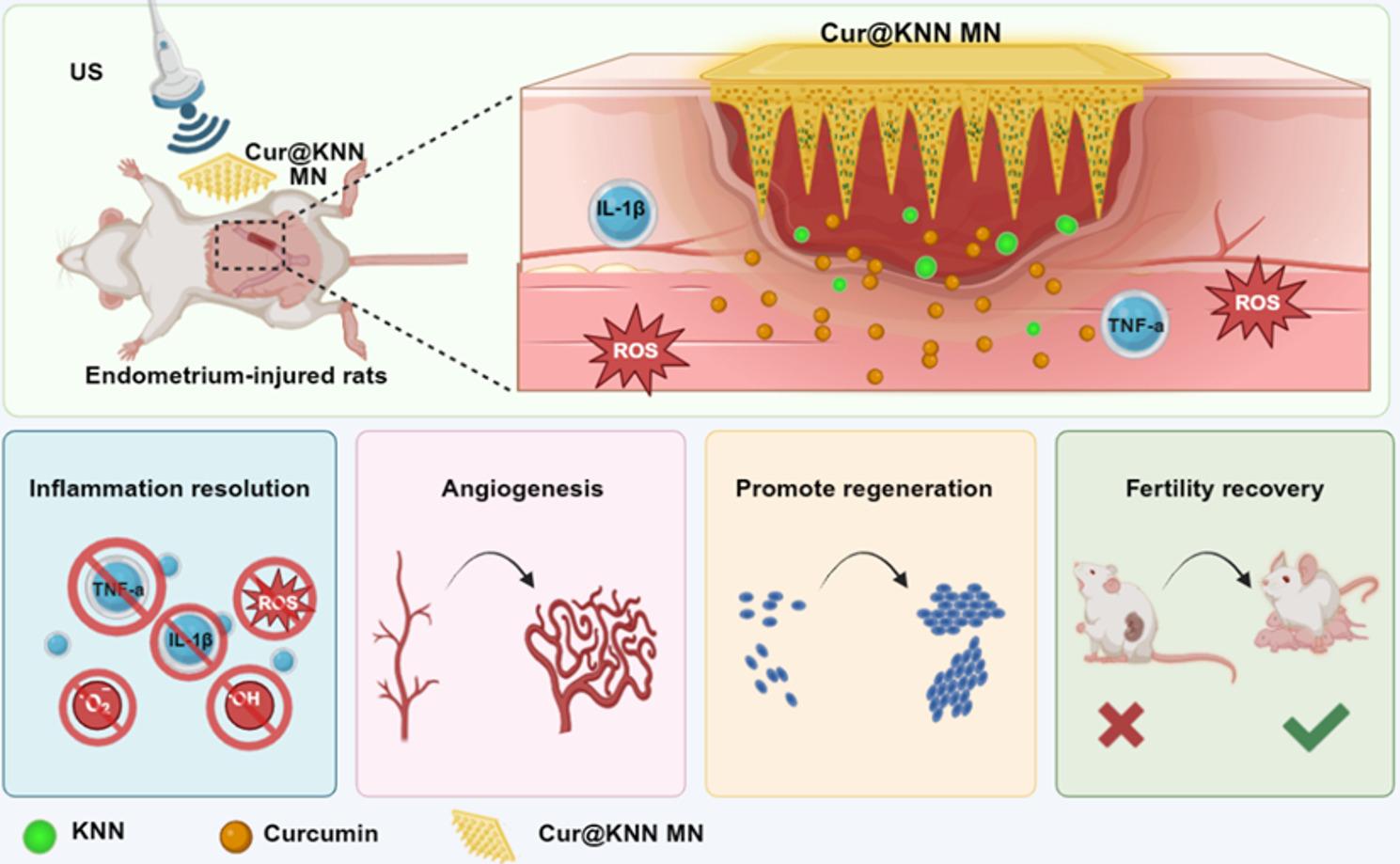

**Supplementary Information:**

The online version contains supplementary material available at 10.1186/s12951-026-04313-5.

## Introduction

The endometrium is essential for embryo implantation and maintaining pregnancy. Basal-layer trauma of the endometrium could lead to scar formation, fibrosis, and intrauterine adhesions, causing menstrual abnormalities, recurrent miscarriages, abnormal placentation, and even infertility [1–4]. In the past few decades, uterine adhesions characterized by endometrial fibrosis and thin uterus caused by curettage and other uterine procedures have posed a serious threat to women’s reproductive health [5, 6]. Conventional therapeutic interventions, such as hysteroscopic adhesiolysis, sequential estrogen therapy, intrauterine devices or balloon stents, and platelet-rich plasma perfusion, can partially restore uterine cavity morphology^7^. However, endometrial repair is a complex and tightly regulated process that is frequently hindered by post-operative infection and inflammatory cascades, posing significant clinical challenges [8]. Moreover, these traditional approaches are still insufficient in dynamically regulating immune microenvironment and promoting robust angiogenesis, and achieving functional basal layer synergistic regeneration, leading to limited pregnancy outcomes and long-term prognosis [9, 10]. Recently, Wireless repair has shown remarkable therapeutic potential in a range of tissue-repair fields, utilizing non-contact, remotely controllable physical energies (e.g., ultrasound, magnetic fields, light, or radiofrequency) to trigger or regulate the treatment process, thereby achieving precise control of wound repair and functional restoration [11, 12]. Therefore, combining the wireless repair strategy with therapeutic drug is expected to break the bottleneck of traditional therapies and provide a minimally invasive, controllable, and safe method for endometrial recovery, which is considered a highly desirable strategy.

**Fig. 1:**
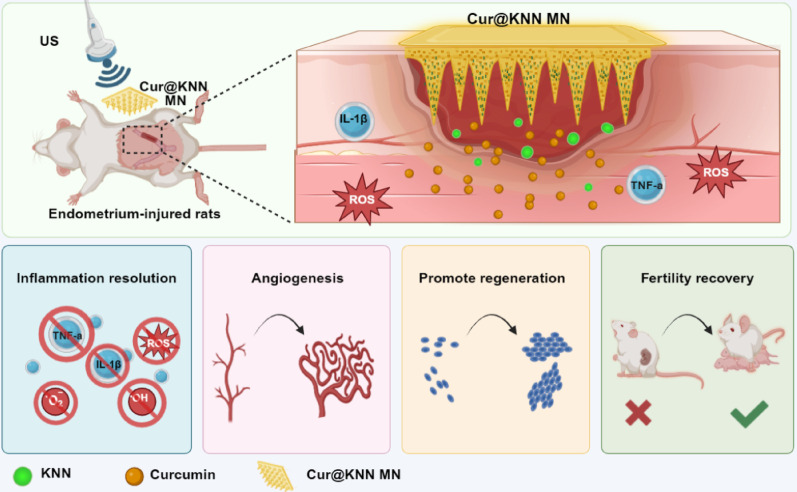
Schematic illustrating Cur@KNN MN patches for wireless endometrial repair. The obtained Cur@KNN MNs penetrate the injured endometrium in a minimally invasive, painless, invasive, and infectious manner. Under the action of ultrasound, curcumin and KNN are continuously released to remove excess inflammatory factors and reactive oxygen species (ROS), promote neovascularization, promote endometrial regeneration, and enhance endometrial receptivity. Cur, curcumin; KNN, potassium-sodium niobate; MNs, microneedles

Herein, we present a novel ultrasound-responsive microneedle (MN) patch that integrates traditional Chinese medicine (TCM) for wireless endometrial repair, as shown in Fig. 1. As an emerging transdermal drug-delivery method, MNs have been proven to penetrate the functional layer and reach the basal layer in a minimally invasive, painless, and low-infection-risk manner [13–17]. Although many active ingredients of TCM exhibit promising therapeutic efficacy in clinical, their clinical translation is severely hampered by poor water solubility, limited stability, and low bioavailability [18]. MNs have unique microscale solid structures and programmable material matrix, which can achieve local, sustained, and controllable release, providing an ideal platform for TCM delivery. Ultrasound (US) therapy, a non-invasive physical stimulus, has received widespread attention due to its deep-tissue penetration, precise spatiotemporal control, and negligible systemic side effects [19]. Over the past decade, coupling US with piezoelectric nanomaterials has been widely applied in thrombolysis, neuromodulation, tumor ablation, and targeted drug delivery [20–25]. Based on these advantages and properties, the integration of MNs and US has been recognized as an intelligent tissue-repair strategy and becomes a new frontier in regenerative medicine. To our knowledge, no prior study has reported the combined use of TCM-loaded MNs and ultrasound for endometrial repair, rendering this approach a promising yet largely unexplored therapeutic avenue.

In this study, we encapsulated piezoelectric potassium sodium niobate (K_0.5_Na_0.5_NbO_3_, KNN) and curcumin (Cur) together into photopolymerized hydrogel to construct an ultrasonic-responsive piezoelectric MN patch (Cur@KNN) for endometrial repair. The patch exhibits excellent biocompatibility and bioactivity. When stimulated by ultrasound, it generates controllable piezoelectric signals and coordinates the sustained release of curcumin, yielding multifaceted therapeutic effects. In vitro, macrophages treated with Cur@KNN demonstrate robust reactive oxygen species scavenging ability and marked anti-inflammatory effects. Vascular-on-a-chip assays further reveal that system accelerates cell proliferation, migration, and angiogenesis, effectively orchestrating tissue regeneration. In a rat model of endometrial injury, Cur@KNN combined with ultrasound therapy can rapidly suppress inflammation, markedly promote the regeneration of smooth muscle and blood vessels in the damaged endometrium, and increase endometrial thickness. Notably, the repaired endometrium supports the normal embryo implantation and development, confirming successful morphological and functional restoration. These findings indicate that this traditional Chinese herb-based piezoelectric MN patches holds significant promise for tissue engineering and regenerative medicine applications.

## Results and discussion

**Fig. 2:**
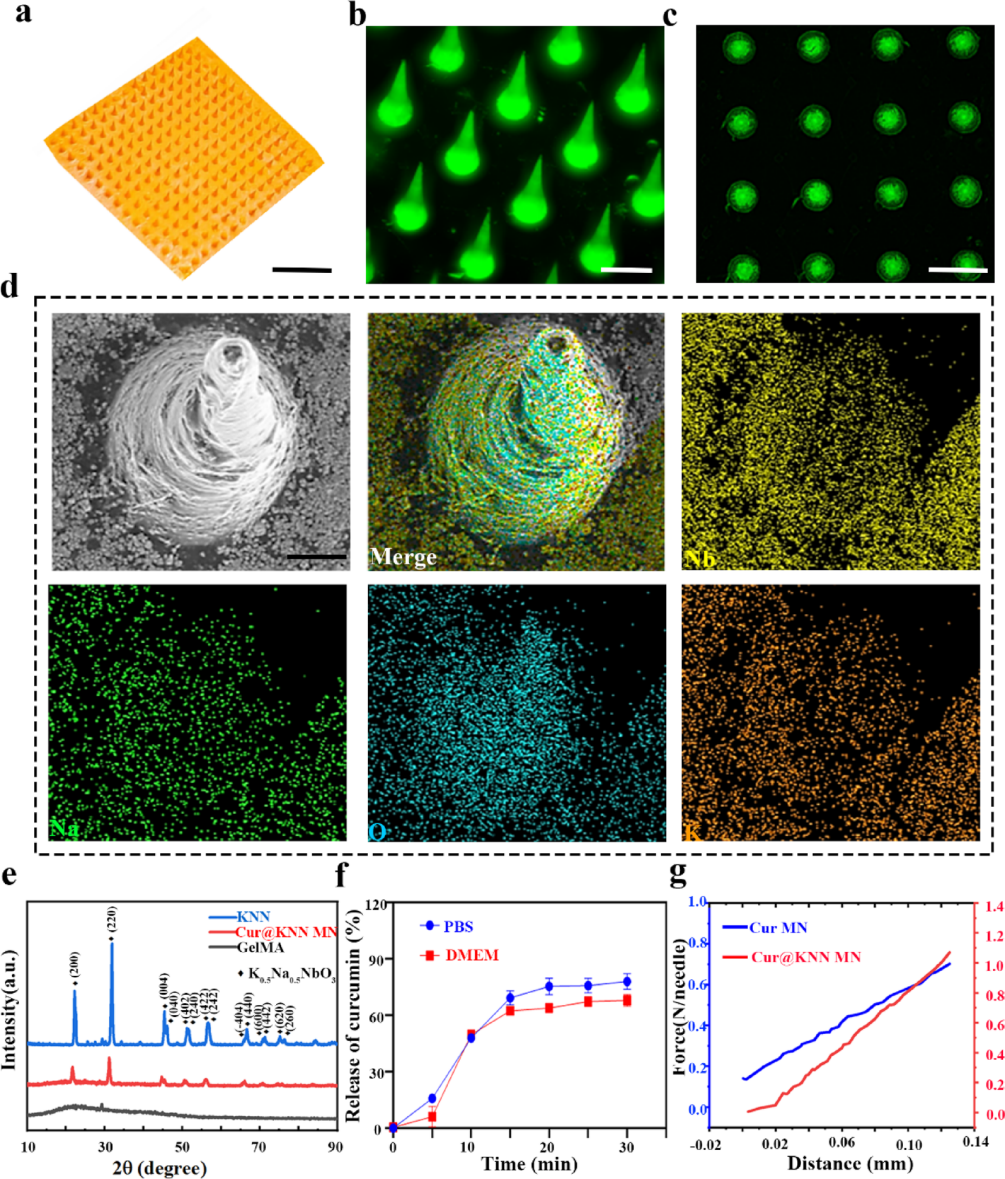
Characterization of Cur@KNN MNs. **a**, The bright field image of Cur@KNN MNs. Scale bars, 3.5 mm. **b-c**, Fluorescence micrograph of Cur@KNN MNs patches. Scale bars, 700 μm. **d**, Elemental mapping showing the composition and distribution of Nb, K, O and Na elements in the Cur@KNN MNs. Scale bars, 100 μm. **e**, The XRD patterns of the KNN, Cur@KNN MNs and GelMA. **f**, Plot of curcumin release from Cur@KNN MNs patch in PBS and DMEM over time. **g**, Force-displacement curves of different patch samples

In a typical experiment, the Chinese herbal piezoelectric MN patch was fabricated by replicating from a negative mold. The negative mold was designed with an ordered array of conical cavities, with depth of 820 μm and a base diameter of 340 μm. GelMA hydrogel was adopted as MN base liquid due to its excellent biocompatibility, biodegradability, and bioactivity [26, 27]. The KNN composed of nontoxic potassium, sodium, and niobium is a dissolvable inorganic material with excellent piezoelectric properties [28]. KNN and curcumin were loaded onto the tip and back layer of MN. Briefly, curcumin and KNN were separately added in GelMA hydrogel solutions. This blend was introduced into the MN negative mold and subjected to repeated vacuum cycles to ensure complete filling. Following this, the structure was cross-linked and solidified under the 405 nm UV light. After drying and demolding, the complete Cur@KNN MN patch was obtained (Figure S1a). By observing the patch, it could be seen that a 15 × 15 array of sharply tapered microneedles uniformly arranged on the backing layer (Fig. 2a), which significantly augmenting their ability to easily pierce the skin. Scanning electron microscopy (SEM) also confirmed the conical structure of the needle tips perched atop the substrate layer (Figure S1c). To better visualize MN structure, fluorescently labeled protein was incorporated into the MN; confocal microscopy revealed homogeneous fluorescence distribution, attesting to uniform tip geometry and well-defined contours (Fig. 2b-c). The KNN distributed uniformly in Cur@KNN MN patch was also confirmed by energy-dispersive X-ray spectroscopy (EDS) mapping of Nb, K, O, Na elements (Fig. 2d). The crystallinity of Cur@KNN MN was further evaluated by X-Ray Diffraction (XRD). Characteristic peaks corresponding to KNN nanowires, such as (200), (111), (220), (004), and (402), were clearly observed (Fig. 2e), confirming the successful integration of KNN into the Cur@KNN MN patch.

The viscoelastic properties of tissue attenuate MNs penetration, necessitating sufficient mechanical strength as a prerequisite for effective insertion [29, 30]. The compression test showed that the MNs in this study exhibited excellent mechanical properties and can withstand pressures exceeding 1.207 N/needle without fracture (Fig. 2g), thereby confirming adequate mechanical strength for penetrating skin and deep tissue layers. In vitro puncture tests further validated that nearly all needles were found to create tiny holes (figure S2b). In addition, the MN-penetrated tissue was further embedded and frozen sectioned, and it was found that MN successfully penetrated the endometrial tissue, achieving a penetration depth of 260–350 μm, which is sufficient to meet the needs of local drug delivery (figure S2c). In terms of degradation characteristics, degradation experiments conducted under simulated uterine conditions (37 °C) revealed that the needles began to dissolve within 20 min and completely disintegrated within 1 h, ensuring natural elimination without surgical removal following drug release (Figure S2d).

The adaptability of microneedle patches in dynamic tissue environments is also a key characteristic. The MNs in this study demonstrated favorable tissue adhesion and morphological adaptability. As illustrated in Figure S3, MNs achieve initial firm attachment via mechanical anchoring effect after microneedling into the tissue. Even under substantial tissue deformation, including lifting, bending, twisting, and stretching ex vivo, the patch remained tightly adherent without detachment. Furthermore, under the synergistic effects of tissue fluid penetration and physiological temperature (37 °C in vitro), the microneedle substrate gradually swelled to form a hydrogel, which enhanced interfacial adhesion through conformal contact. This enabled the patch to adapt to secondary tissue deformation while maintaining stable fixation (Figure S3), demonstrating excellent shape-adaptive retention, ensuring stable retention throughout the entire administration period.

Subsequently, to evaluate the release kinetics of curcumin, MN patches were submerged in PBS and DMEM. Draw the release curve by monitoring the absorbance of the supernatant at 430 nm. Curcumin attained release saturation within 30 min, with cumulative release rates of 77.99% in PBS and 67.92% in DMEM, respectively (Fig. 2f), which confirming favorable drug-release characteristics of the patch. However, it is worth noting that there is an essential difference between the conditions of complete dissolution in vitro and the actual environment in vivo. In uterine tissue, due to the limited amount of local exudation, the microneedles only change to the hydrogel state rather than complete liquefaction, so as to achieve the continuous osmotic release of drugs. Ultrasonic stimulation can further regulate this process. In the in vitro release experiment of this study, as the ultrasonic intensity increased, the drug release rate significantly accelerated (Fig. 4f), indicating that the piezoelectric effect activated by ultrasound can promote drug diffusion from the MN. This may be attributed to the micro-electric field generated by the piezoelectric effect, which facilitates drug diffusion from the microneedle matrix; and the electroosmotic effect promotes water migration, indirectly affecting the swelling behavior of the microneedles.

**Fig. 3:**
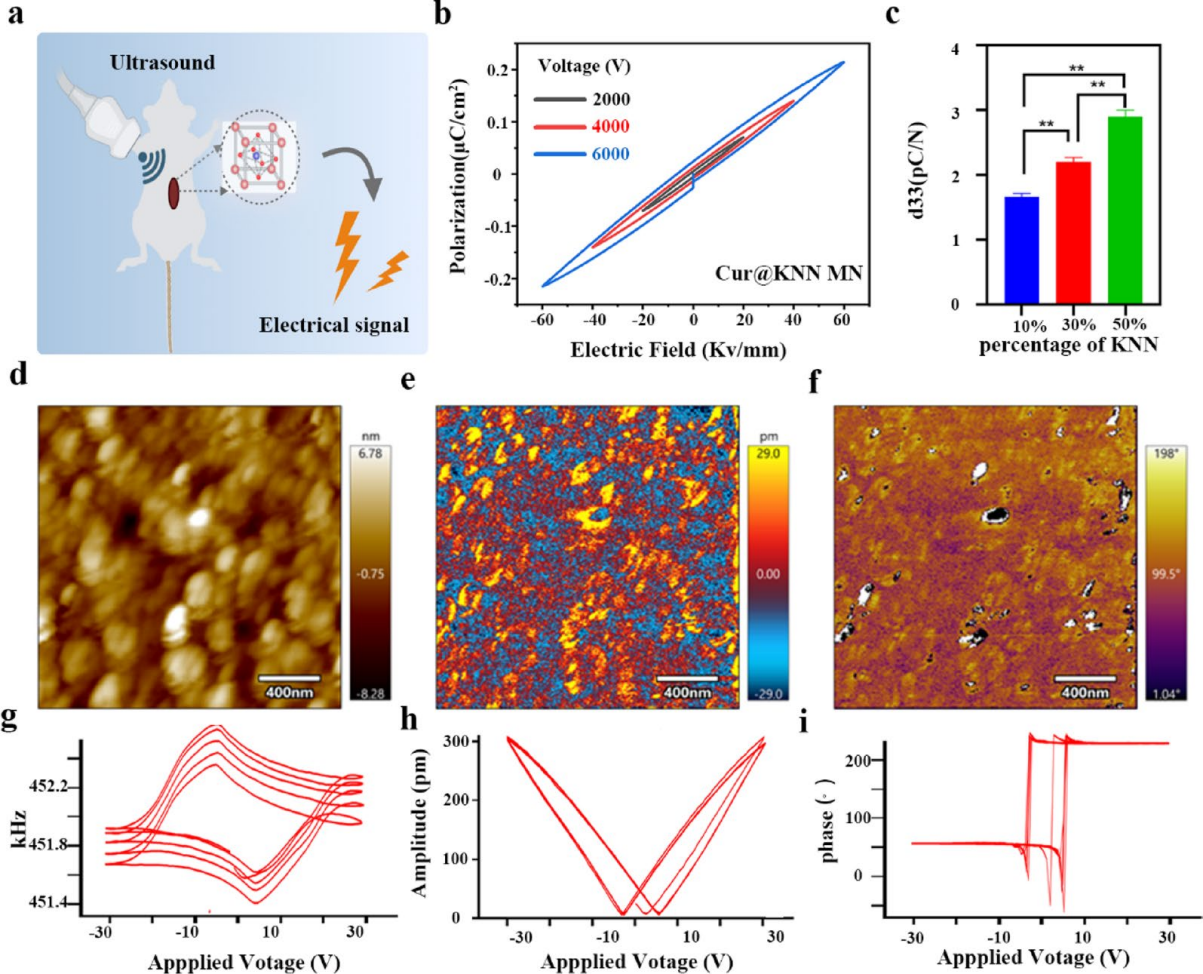
**a**, Schematic diagram depicting the Cur@KNN MNs-mediated piezoelectric effect. **b**, The polarization hysteresis loops of Cur@KNN MN. **c**, The piezoelectric coefficient (d33) of 10%, 30%, 50% Cur@KNN MN. **d**, Evaluation of surface morphology via piezoresponse force microscopy (PFM). **e**, Evaluation of amplitude via PFM. **f**, Evaluation of phase via PFM. **g**, Frequency of the PFM signal of Cur@KNN MN. **h**, Amplitude of the PFM signal of Cur@KNN MN. **i**, Phase of the PFM signal of Cur@KNN MN at room temperature

To gain a deeper understanding of this regulatory mechanism, this study systematically characterized the piezoelectric properties of Cur@KNN MN. When piezoelectric materials are subjected to ultrasonic response, the lattice structure undergoes deformation, resulting in charge redistribution and the generation of a potential difference, thereby effectively converting mechanical energy into chemical energy (Fig. 3a). Firstly, use a ferroelectric analyzer to record the hysteresis loop of the material. As shown in Fig. 3b, with increasing applied voltage, the hysteresis curve gradually becomes apparent, indicating favorable piezoelectric performance. The d_33_ piezoelectric coefficient is a key parameter that describes ability of a material to generate electric charge under mechanical stress. A higher d_33_ value corresponds to a stronger piezoelectric effect, enabling greater charge output and higher sensitivity. In this study, the d_33_ value of the polarized Cur@KNN MN patch was measured using a quasi- static d_33_ m (ZJ-3AN). The d_33_ value of the 10% Cur@KNN was 1.7 pC/N, whereas that of the 50% Cur@KNN reached 2.9 pC/N (Fig. 3c). This increase in d_33_ with higher KNN mass ratio demonstrated a significant enhancement in piezoelectric output performance. In addition, the piezoelectric performance of Cur@KNN MN was further characterized using piezoresponse force microscopy (PFM), a widely employed technique for analyzing piezoelectric materials. The Cur@KNN MN exhibited a notable piezoelectric response under alternating electric fields, as evidenced by the amplitude and phase signals (Fig. 3d-f). A characteristic butterfly-shaped amplitude curve was observed in the piezoresponse amplitude under a ramp voltage from −30 to +30 V, indicating constant strain variation induced by the external electric field (Fig. 3h). Furthermore, the local piezoelectric hysteresis loop in the phase diagram exhibited a phase shift of ≈ 300°, further indicating the intrinsic piezoelectricity of the Cur@KNN MN (Fig. 3^3^). Together, these results highlight the excellent piezoelectric performance of the Cur@KNN MN system.

**Fig. 4:**
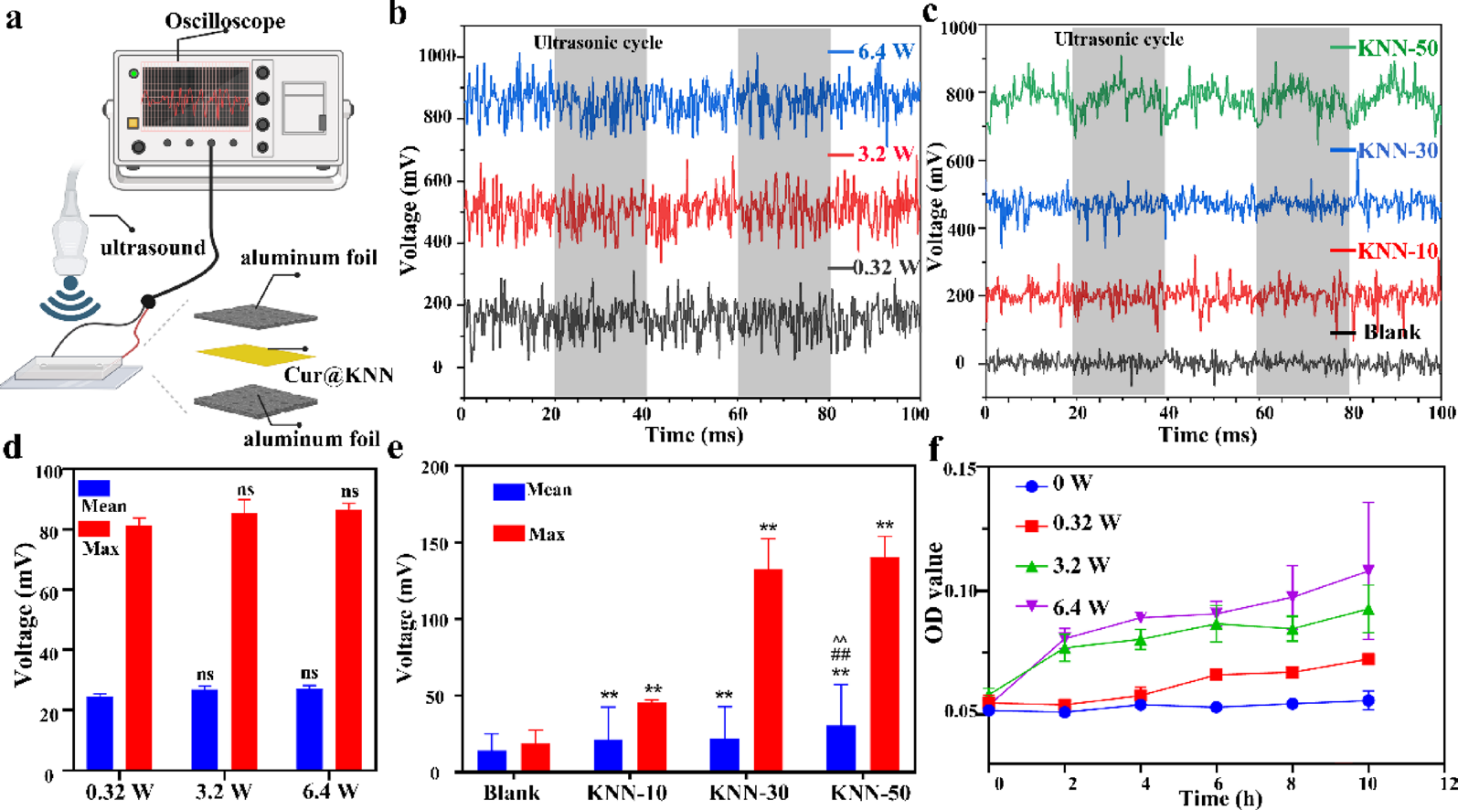
Piezoelectric MN responses to the LIFU. **a**, Schematic diagram of testing setup used to evaluate the electrical output of piezoelectric MN under LIFU. **b**, The electrical output at different output power levels detected by the oscilloscope. **c**, The electrical output response to 1 MHz LIFU of the KNN-50, KNN-30, KNN-10 and blank control detected by oscilloscope. **d-e**, The electrical output range of different output power levels and different concentrations of KNN. **f**, Drug release under different output powers. * *P* < 0.05, ** *P* < 0.01. The asterisks (*) indicated their respective comparison with the blank group

Based on the excellent piezoelectric properties of MN, this study further evaluated the electrical output behavior of MNs under low-intensity focused ultrasound (LIFU) exposure. The Cur@KNN hydrogel was cast into a 1 × 1 cm square sheet, with both sides clamped by aluminum foil electrodes to form a sandwich structure. The electrical signals under ultrasonic stimulation were collected using an oscilloscope (Fig. 4a). When exposed to 1 MHz focused ultrasound (50% duty cycle), the voltage output differences under different ultrasonic output power were not significant (Fig. 4b). Both the maximum output voltage and the average output voltage showed no statistical significance between groups (Fig. 4d). However, the concentration of KNN had a significant impact on the output. The electrical outputs of KNN-50 (maximum output 140.2 mV, average 30.46 mV) and KNN-30 (maximum output 132.4 mV, average 22.03 mV) were significantly higher than that of KNN-10 (maximum output 45.62 mV, average 21.34 mV) (Fig. 4c, e). These results indicate that the aggregation structure of KNN nanoparticles in the MN significantly enhances the ultrasonic-triggered piezoelectric output, providing a physical basis for achieving drug-controlled release and bioelectrical stimulation under ultrasonic mediation.

**Fig. 5:**
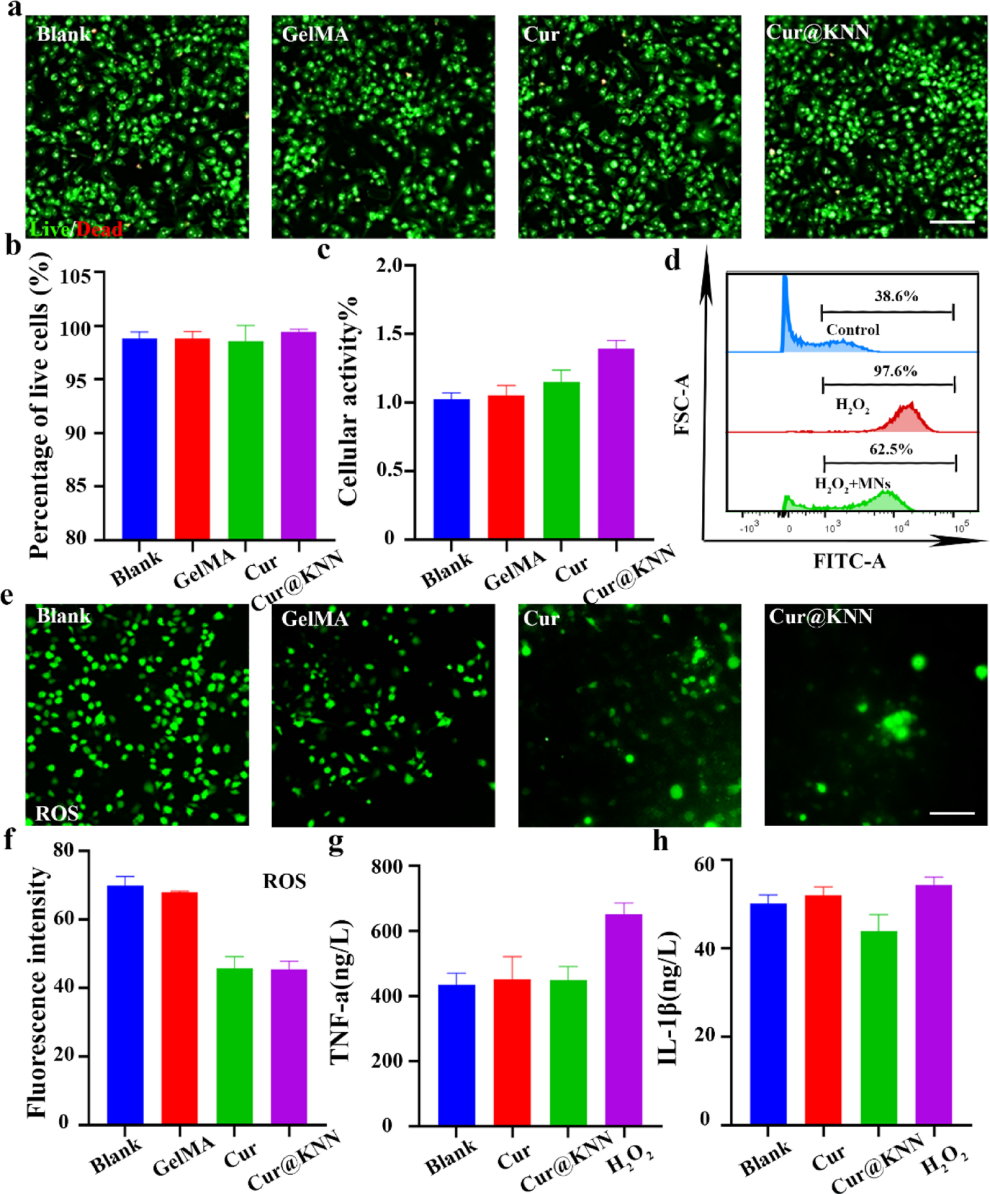
Characterization of biocompatibility and anti-inflammatory effects of the Cur@KNN MNs. **a**, Live/dead cell assay after treatment with nanozyme, live cell (green), dead cell (red), scale bar, 100 μm. **b**, Cell survival rate analysis. **c**, CCK-8 assay after different treatments. **d**, FACS analysis for ROS generation in macrophages with exposure to MNs. **e**, ROS fluorescence staining. Scale bars, 100 μm. **f**, Quantitative analysis of the ROS signal after treatment. g-h, ELISA assay for the secretion of inflammatory factors TNF-a (**g**) and IL-1β (**h**) in cell supernatant with exposure to curcumin and Cur@KNN MNs. At least three images were randomly selected from each group for quantification

To further systematically evaluate the cytocompatibility of MN, cell viability was performed using the live/dead cell assay. Three groups were established: GelMA group, Cur group, Cur@KNN MN group, and PBS (blank) group, co-cultured with RAW cell as the model. As shown in Fig. 5a, the cell viability staining after 24 h of RAW culture revealed that the proportion of live cells in the three groups was still high. These values showed no statistically significant difference in the ratio compared with blank group (*P* > 0.05) (Fig. 5b), which indicated that both materials remained no-cytotoxic at seeding. Notably, the trends of differentiation and proliferation were observed after 48 h (*P* < 0.05) (Fig. 5c). This phenomenon confirms that the MN matrix has highly biocompatible, and loading Cur and KNN does not affect its safety. Cur@KNN MN promotes RAW cell proliferation through controlled release of curcumin. Additionally, to verify the in vivo biocompatibility of MN, the Cur@KNN MNs were implanted into the uterine tissue of SD rats to investigate. After 2 weeks, various organ tissues were taken for histological analysis. No abnormalities were found in H&E staining (Figure S7). All results indicate that Cur@KNN MNs could be safely used for in vivo applications without evident immune rejection and inflammatory reactions.

To recapitulate an inflammatory environment in vitro, H₂O₂ (1 mM) was added to macrophage culture medium. Subsequently, we subjected the macrophages to treatments with curcumin and Cur@KNN MN extract-infused medium, closely monitoring their inflammatory phenotypes. Flow-cytometric analysis revealed that the proportion of ROS from 97.6% (H₂O₂ group) to 62.5% within the H₂O₂ + MNs group (Fig. 5d), indicating a synergistic anti-inflammatory effect. Fluorescence staining revealed that macrophages stained with DCFH-DA (green) showed stronger fluorescence staining in the H₂O₂ group, while after intervention with MNs, the fluorescence signal was weakened (Fig. 5e). Further statistical analysis of ROS levels showed that the H₂O₂ group exhibited enhanced ROS signaling, while MN significantly reduced ROS (Fig. 5f). Culture supernatant assays demonstrated that H₂O₂ markedly elevated pro-inflammatory cytokines (TNF-α and IL-1β), whereas curcumin and MN reversed these elevations (Fig. 5g-h). ELISA further corroborated the suppressive action of MN against pro-inflammatory stimuli, which may be attributed to the synergistic enhancement of KNN nanoparticles. KNN possesses piezoelectric properties, and ultrasound-triggered piezoelectric responses not only promote cell proliferation and enhance cellular uptake, thereby potentiating the therapeutic efficacy. In summary, these results indicate that MN exerts its anti-inflammatory activity by modulating the ROS signaling axis.

**Fig. 6:**
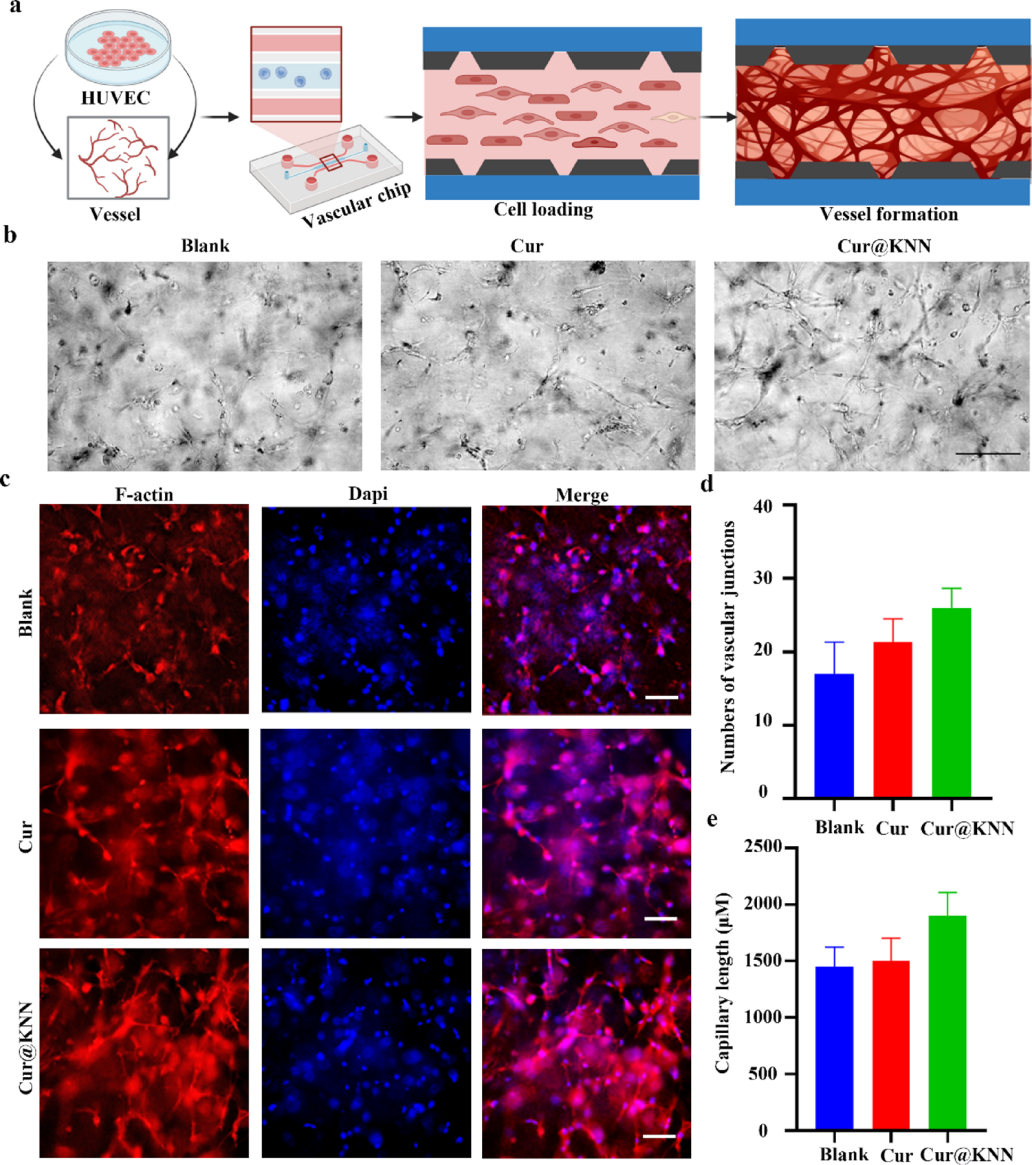
The impact of Cur@KNN MNs on tube formation. **a**, The model diagram of vascular chip and the schematic diagram of the vessel formation. **b**, Representative phase contrast micrographs of microvascular networks formation in microfluidic devices at different intervention treatments. Scale bars, 200 μm. **c**, Fluorescence microvascular networks images expressing F-actin. Scale bars, 100 μm. **d-e**, Quantification of number of vascular junctions and capillary length

New blood vessel is indispensable for the generation of any tissue, and microvascular reconstruction is therefore a prerequisite for endometrial regeneration. In this study, the ability to promote angiogenesis of Cur@KNN MN was comprehensively evaluated in vitro using microvessel-on-a-chip. The model diagram of vascular chip is shown in Fig. 6a. By using different processed human umbilical vein endothelial cells (HUVECs) culture media for vascular chip co culture. The results showed that HUVECs from the MN group exhibited enhanced proliferative activity and tube-forming capacity, suggesting that MN contributes to the restoration of functional vascular networks (Fig. 6b). Using the ImageJ vascular generation analyzer plugin to quantify the number of vascular connections and capillary length, the results showed that MN connections had more connections and longer capillary lengths (Fig. 6d-e). For further visualization, immunofluorescence staining was performed on the vascular chip. As shown in the Fig. 6c, compared with the blank group, F-actin fluorescence rose markedly after MN treatment, indicating that MN can promote vascular formation. Similarly, 2D Tube formation assay has also confirmed that MN accelerated HUVEC tube formation (Figure S4a). The results of quantifying the number of vascular connections and capillary length are consistent with the above trend (Figure S4c-d). Furthermore, a cell-scratch assay was employed to assess chemotaxis and wound-repair capacity. The representative images of wound healing tests indicate that the MN group heals faster (Figure S4b). The MN group exhibited a significantly higher scratch healing rate than both the control and curcumin groups, underscoring its superior migratory and reparative effects (Figure S4e). Previous studies have shown that curcumin complexes can exert endothelial cell protection by regulating the expression of apoptosis related genes, thereby promoting angiogenesis during wound healing and maintaining a stable balance of angiogenesis in the wound microenvironment. In this study, we further validated the expression changes of the mechanism related proteins through Western blot experiments. The results showed that compared with the model group, the expression levels of angiogenesis marker CD31 and anti-apoptotic protein Bcl-2 were significantly increased in the Cur/Cur@KNN group (Figure S5 a-c). This result suggests that curcumin complexes may exert repair effects through a dual mechanism of activating angiogenesis and inhibiting cell apoptosis. Overall, these data demonstrate, Cur@KNN MN promoted the function of vascular endothelial cells and facilitated tissue repair processes in wound healing.

**Fig. 7:**
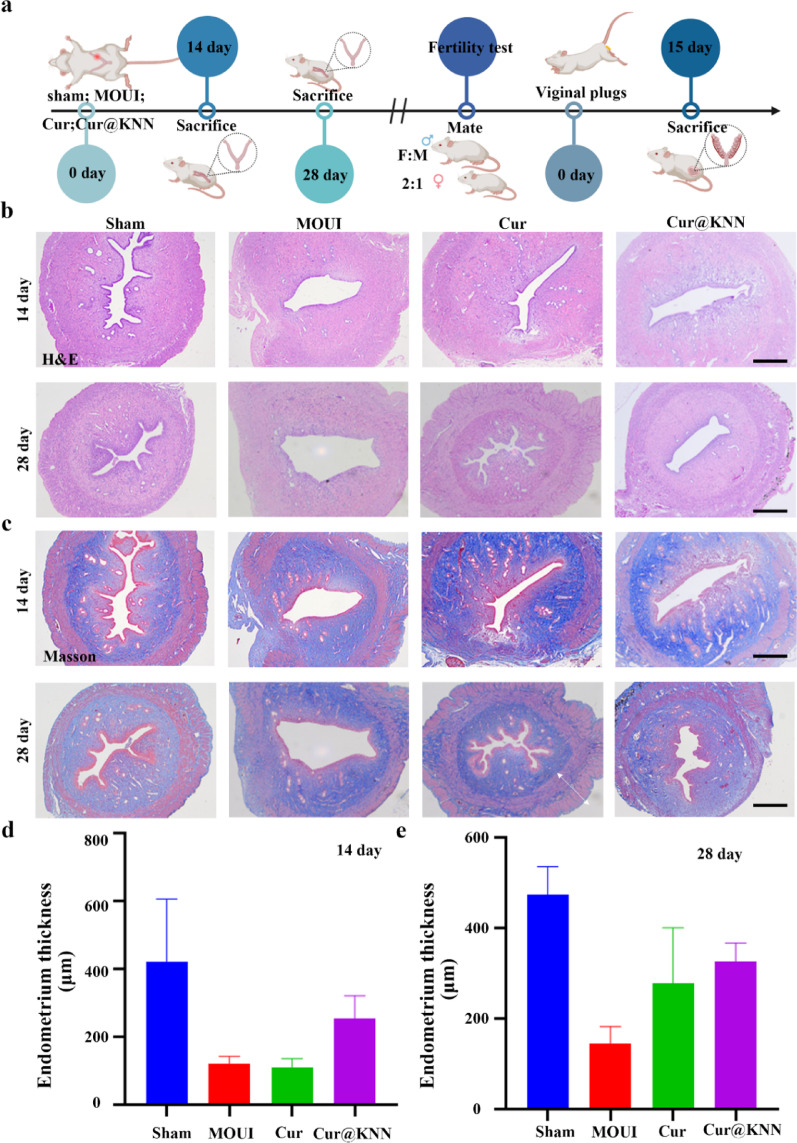
The pathological changes after the treatment of Cur/MN. **a**, In-vivo schedule. b-c, H&E staining of the uterus at 14 (**b**) and 28 (**c**) days in sham group, models of uterine injury (MOUI) group, curcumin (Cur) group and Cur@KNN MN group. Scale bar, 400 μm. d-e, Quantitative analysis of endometrium thickness at 14 (**d**) and 28 (**e**) days. Data were presented as mean ± SEM

Given the multifaceted bioactivity of Cur@KNN MN observed in vitro, we next evaluated its repair capacity in vivo by a rat model of endometrial injury. Specifically, cut a notch on the right side of each uterine horn and gently scrape the exposed surface. Then Cur or Cur@KNN MN patches were attached to the uterus, whereas the sham group underwent laparotomy only. After suturing, the Cur@KNN MNs group received transabdominal low-intensity focused ultrasound (LIFU) treatment. According to previous research, this ultrasound parameter (1 MHz) can achieve a tissue penetration depth of approximately 5 cm, ensuring effective drug delivery to the target tissue. In addition, the mechanical effect of ultrasound can promote changes in cell function, improve blood and lymphatic circulation, enhance cell membrane permeability, thereby improving metabolic levels and tissue regeneration ability [31]. At the same time, it can soften connective tissue and has been clinically used to treat scars, adhesions, and scleroderma. It is worth noting that the ultrasound equipment used in this study has an overheat protection mechanism, and most of the heat generated is lost through blood circulation, while the rest is dispersed to surrounding tissues through thermal conduction, which can avoid irreversible tissue damage. Finally, the rats in each group were euthanized on postoperative days 14 and 28 to evaluate the effectiveness of uterine repair (Fig. 7a).

Macroscopically, the appearances of uterine horns in MOUI and treatment groups were more similar, which the damaged gap mixed with the regenerated tissue (Figure S6). H&E staining revealed that Cur@KNN MN treatment presented well-formed morphology within 14 days, including a significantly thicker regenerated endometrium compared with MOUI group (Fig. 7b and d). Moreover, the Cur@KNN MN group displayed a continuous, well-organized myometrium, whereas smooth-muscle disruption was broken at the injured sites in MOUI groups. After 28-day treatment, the endometrial thickness in the Cur@KNN MN group reached 325.27 ± 42.18 μm, remarkably thicker than that in the MOUI (144.5 ± 38.48 μm; *P* < 0.0001) groups, and showed no significant difference with that in sham group (Fig. 7e). Furthermore, Masson staining further demonstrated extensive collagen deposition in the MOUI group, indicative of poor prognosis (Fig. 7c). In contrast, Cur@KNN MN treatment reduced fibrosis, yielding a collagen density and alignment comparable to those of the sham group.

**Fig. 8:**
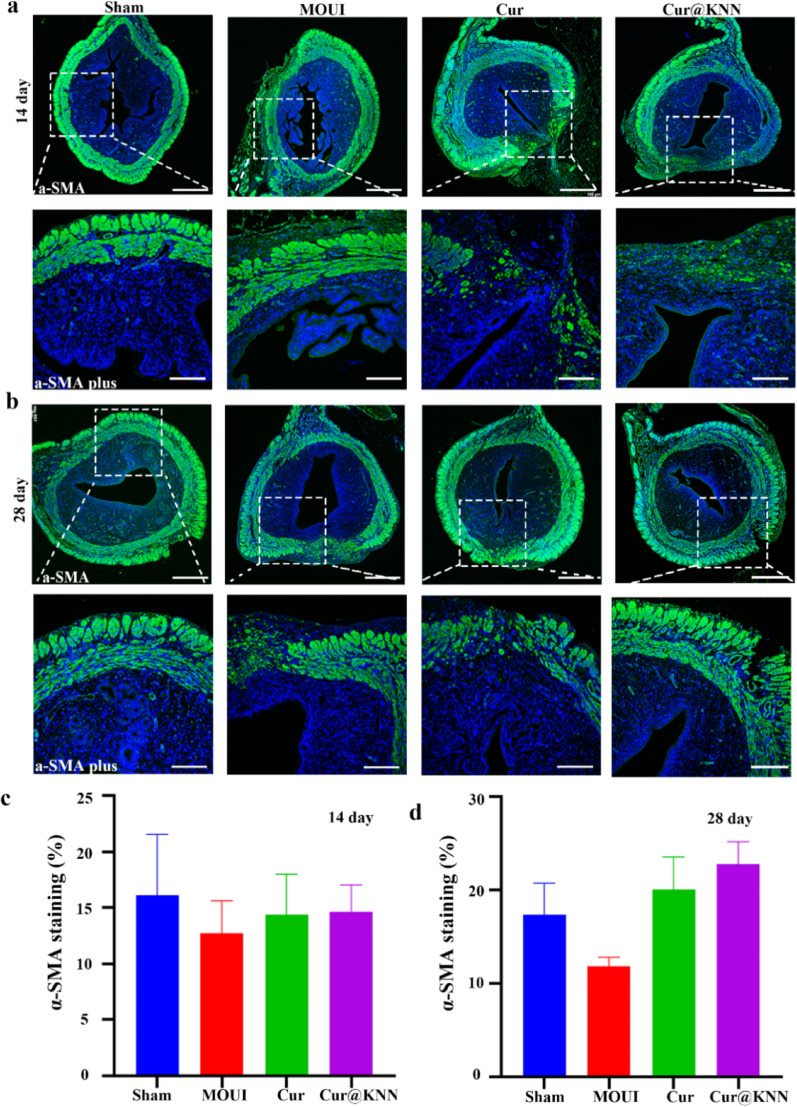
The regeneration of smooth muscle after the transplantation of Cur@KNN MNs. Immunofluorescence staining of α-SMA for smooth muscle at 14 (**a**) and 28 (**b**) days in sham group, models of uterine injury (MOUI) group, curcumin (Cur) group and Cur@KNN microneedles (Cur@KNN) group (a and b). Scale bars, 500 μm (up), 200 μm (down). Quantitative analysis of α-SMA (%) at 14 (**c**) and 28 (**d**) days. Data were presented as mean ± SEM

The myometrium, composed of smooth-muscle cells, is indispensable for maintaining uterine integrity [32]. Therefore, we examined smooth-muscle regeneration at the 14th and 28th day post-surgery. Immunostaining of α-SMA showed that Cur/Cur@KNN groups displayed newly formed a continuous loop at 14th day, in stark contrast to the interrupted muscular layer observed in the MOUI group at 14th day (Fig. 8a). Quantitative analysis showed that the percentage of α-SMA staining in the Cur/Cur@KNN groups (14.40 ± 3.59%, 14.65 ± 2.38%) were higher than in the MOUI over the same period (12.77 ± 2.86%) (Fig. 8c). Moreover, the myometrium in the MN group had become markedly smoother and more like normal tissue (Fig. 8b), with abundant, well-aligned muscle bundles evident throughout the repaired segment. The percentage of α-SMA in the Cur/MN group (20.11 ± 3.43%, 22.76 ± 2.43%) remained significantly higher compared with MOUI group (11.8 ± 1.0%) (Fig. 8d). Collectively, these data demonstrate that Chinese herbal piezoelectric MN patches effectively promote the regeneration of uterine smooth muscle.

**Fig. 9:**
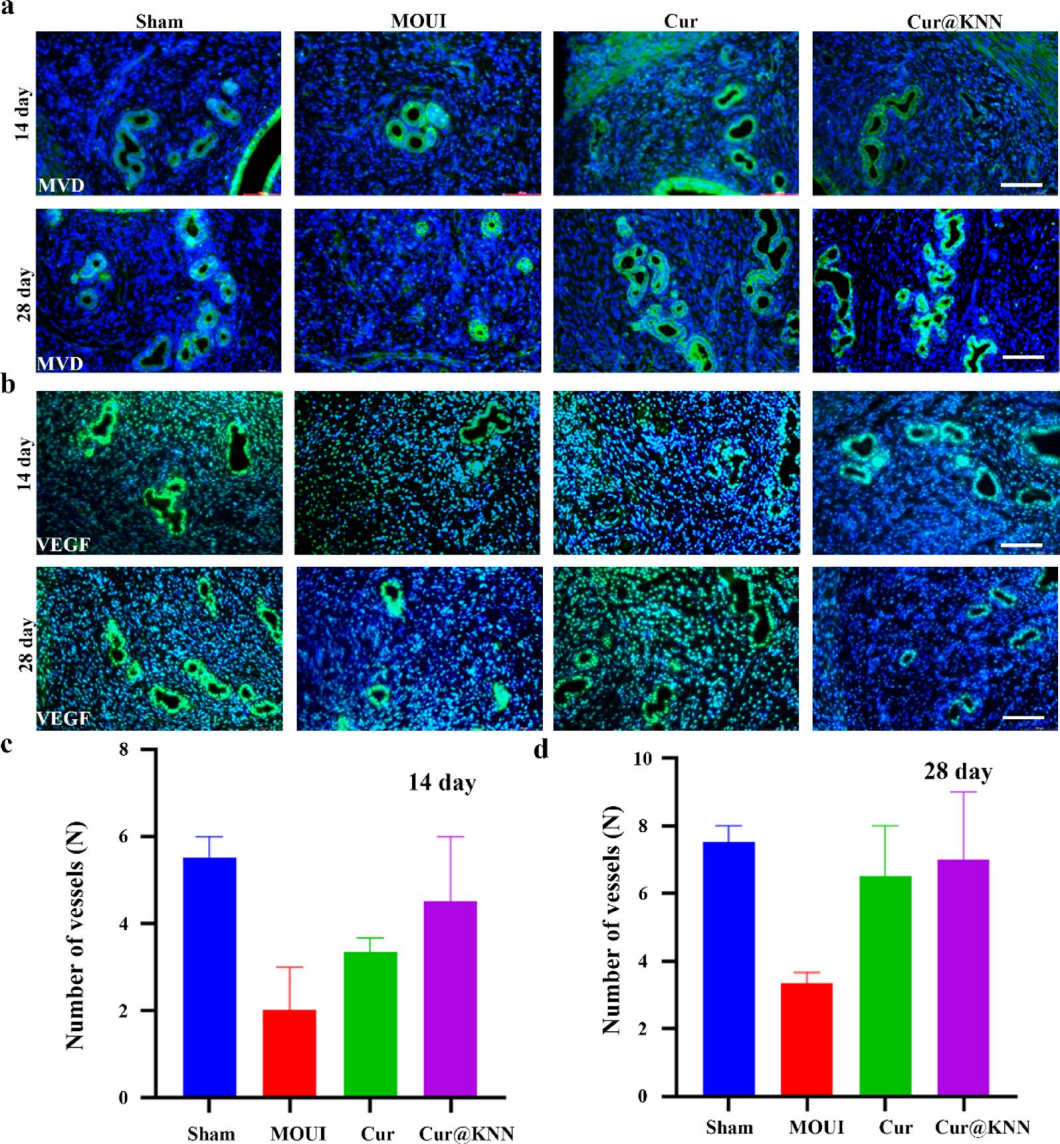
Cur@KNN MN treatment accelerated angiogenesis. MVD (**a**) and VEGF (**b**) immunofluorescence at 14 and 28 **d**. Scale bar, 100 μm. Quantitative analysis of the numbers of vessels. Data were presented as mean ± SEM. *P < 0.05, **P < 0.001

Angiogenesis is essential for the functional recovery of the endometrium [33]. Therefore, we evaluated neovascularization at the injured uterine sites by immunofluorescence staining of VEGF and MVD. The MN group exhibited robust revascularization within the endometrial functional layer after transplantation for 14th day, while vessels in the model group remained sparse (Fig. 9a). Quantitative counts showed Cur@KNN MN-treated uterus contained 4.5 ± 1.5 vessels, doubling the 2 ± 1 seen with MOUI (*P* < 0.01, Fig. 9c), and by day 28 the vascular density approached that of the sham group (Fig. 9b). The number of blood vessels marked by MVD and VEGF was 7 ± 2 in Cur@KNN MN group, compare with 3.33 ± 0.33 in MOUI (*P* < 0.0001) (Fig. 9d).

**Fig. 10:**
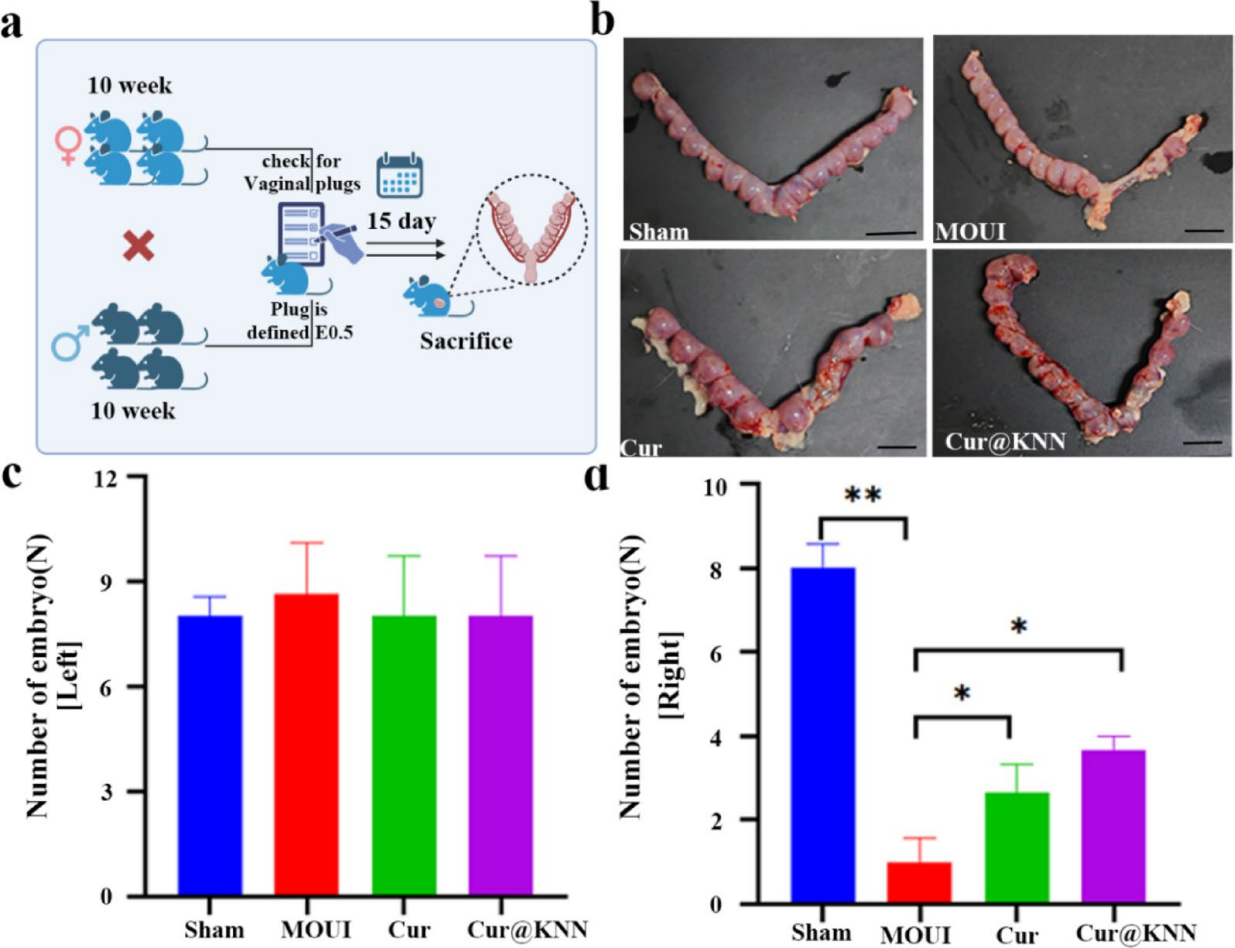
MNs improved fertility recovery. **a**, The schematic diagram of fertility testing. **b**, Images of uterine horns and embryo counts. **c-d**, Number of implantation sites on the uninjured side (left) and injured side (right) of the uterine horn (*n* = 3). Results are presented as mean ± SEM, *P < 0.05, **P < 0.001. Scale bars, 1 cm

Conception rate in rats is an important indicator for successful embryo implantation and evaluation of endometrial receptivity [34]. Consequently, fertility testing was assessed 28 days after intervention. Mate male and female rat in a 1:2 (Fig. 10a). The results showed that under normal pregnancy conditions, the left implantation site of all rats was normal (uninjured) (Fig. 10b-c). In contrast, the right (injured) horns of the MOUI, curcumin, and MN groups displayed restricted implantation, with the most severe impairment observed in the MOUI group. Curcumin and Cur@KNN MN treatments partially rescued endometrial function. Notably, the Cur/Cur@KNN group could hold more embryos alive (2.67 ± 0.67, 3.67 ± 0.33) than the MOUI group (1.00 ± 0.57, *P* < 0.001) (Fig. 10d). Thus, Cur@KNN MN patches loaded with curcumin markedly promoted functional reconstruction of the injured uterus, permitting embryo implantation and establishing an environment conducive to fetal development.

Overall, the piezoelectric-responsive drug delivery system constructed in this study provides an innovative therapeutic strategy for endometrial repair. The system synergistically promotes endometrial repair through the physical signals generated by piezoelectric effect and the chemical signals released by drugs. However, this study still has the following limitations. Firstly, regarding the issue of uterine tissue exposure, the microneedle structure designed in this study aims to achieve drug delivery by encapsulating active ingredients at the tip of the microneedle and penetrating deep into the damaged tissue matrix. However, its puncture process may cause mechanical damage to the basal layer of the endometrium, which may indirectly induce the formation of scar tissue. Secondly, microneedle arrays require invasive implantation into the uterus through open abdominal surgery, which severely limits their clinical applicability. Future research may consider combining microneedles with existing uterine instruments, such as uterine balloons and intrauterine stents to develop non-invasive drug delivery regimens. In addition, although piezoelectric materials have been widely used in medical fields such as tumor treatment, antibacterial, tissue repair and regeneration [35], and biosensing, their degradability, long-term safety, and potential impact on the reproductive system in endometrial tissue are unclear, and there are regulatory approval barriers.

At present, the field of endometrial repair is in a rapid development stage. The breakthrough progress of regenerative medicine and tissue engineering technology, as well as the diversified design of new biomaterials, have opened up a new path for overcoming female reproductive problems [8, 36]. Stem cell therapy, platelet-rich-plasma (PRP), and stem cell exosomes have accumulated a large amount of preclinical and clinical data in the field of regenerative medicine. The active substances released by them, such as cytokines, significantly promote cell proliferation and tissue regeneration, providing new options for endometrial regeneration and patient reproductive function recovery [37, 38]. However, they still have certain limitations: the risk of immune rejection of non-autologous stem cells, the lack of standardized preparation processes for PRP and exosomes, unstable storage conditions, and immature quality control systems all restrict their clinical promotion. This study innovatively applies piezoelectric materials to endometrial repair for the first time. Although it is currently only in the stage of concept validation and in vitro research, the actual efficacy and safety cannot be directly compared with mature therapies. However, the preliminary experimental results are encouraging. In addition, the emergence of new nonlinear microneedles, which have unique curved or branched structures, in recent years can significantly improve tissue penetration depth and drug delivery efficiency [39]. In the future, the advantages of this structure can be integrated to further optimize the design of piezoelectric herbal microneedle systems, promoting their application and development in the field of endometrial repair.

## Conclusions

In this study, we have presented a novel ultrasound-responsive curcumin-loaded MN patch specifically designed for endometrial repair in a rat model. The MN patch was fabricated using GelMA as the matrix, with curcumin and the piezoelectric KNN nanoparticles co-loaded into the needle tips and backing layer. This synergistic combination endows the patch with three unique attributes: antioxidant activity, ultrasound-triggered responsiveness, and sustained drug release. In vitro, MN exerts strong anti-inflammatory and antioxidant effects, which can significantly reduce ROS levels and inflammation caused by H_2_O_2_ stimulation. The microvessel-on-a-chip models further demonstrated that this system promotes cell proliferation, migration, and angiogenesis, effectively orchestrating the tissue regeneration process. The results of animal experiments indicate that Cur@KNN MN intrauterine implantation can significantly increase endometrial thickness, and promote regeneration of endometrial smooth muscle and blood vessels in the damaged area. Most importantly, successful embryo implantation was achieved at the injury site, with a marked increase in the number of live births. In summary, these results indicate that the traditional Chinese medicine (TCM)-based piezoelectric microneedle patch not only contributes to the morphological reconstruction of the damaged uterus but also restores its reproductive function and improves pregnancy rates.

The integration of piezoelectric materials, traditional Chinese medicine, and microneedle technology provides a flexible and precise platform for combined treatment of endometrial injury. The numerous tiny needles allow for the patch to penetrate the endometrial surface, ensuring site-specific delivery of curcumin to the injured site. Meanwhile, the piezoelectric materials within the MNs can convert low-frequency ultrasound into localized microcurrents, synergistically enhancing curcumin release, which allows MN to exhibit potent ROS scavenging, anti-inflammatory, and pro angiogenic properties. Overall, this study establishes an innovative TCM-based piezoelectric microneedle patch as an effective strategy for in suit endometrial repair. By integrating painless, on-demand drug release, localized piezoelectric stimulation, and immune-microenvironment modulation, this strategy offers a paradigm-shifting approach to regenerate the injured endometrium, restore fertility. It is worth noting that this study provides preliminary conceptual validation and in vitro experimental evidence for the application of piezoelectric herbal microneedle system in the field of endometrial repair, but there is still a significant gap from clinical translation. Subsequent research needs to address the key issues mentioned above and further promote technological optimization to advance this technology towards clinical applications.

## Materials and methods

### Materials

Curcumin (C477437) were supplied from Aladdin Chemical (Shanghai). Potassium sodium niobate (K0.5Na0.5NbO3, KNN) were purchased from Qi Jin New Material. 2 hydroxy-2-methylpropiophenone (HMPP) were purchased from Sigma-Aldrich (USA). Methacrylated gelatin (gelMA) was bought from EFL Co., Ltd (Suzhou, China). Alexa-Fluor-488-labelled goat anti-rabbit IgG was purchased from Abcam. α-SMA, MVD, VEGF antibodies were supplied from Proteintech. HUVECs were obtained from Affiliated Hospital of Nantong University. Cells were cultured in Endothelial Cell Medium (ECM, from Jcellm) mixed with 10% (v/v) Fetal Bovine Serum (FBS, from Gibco) and 1% (v/v) penicillin-streptomycin double antibiotics (NCM) at 37 °C, 5% CO₂. Male rats (9–10 weeks) and female Sprague Dawley (SD) rats (4–6 weeks) were provided by the Animal Center of Nantong University. The study was approved by the Review Committee and Research Ethics Committee of Affiliated Hospital of Nantong University.

### Preparation and characterization of Cur@KNN MNs

A 30% (w/v) curcumin, 30% (w/v) KNN, 30% (w/v) GelMA, and 1% (v/v) 2-hydroxy-2-methylpropiophenone (HMPP) aqueous solution was prepared as the MN precursor. This mixture was loaded into a MN negative mold with elongated conical cavities (≈ 820 μm depth, ≈ 340 μm width). After repeated vacuum cycles, remove bubbles with the tip of a 1 mL syringe needle, the mold was then heated at 65 °C for 5 min. Under gravity, KNN nanoparticles selectively sedimented to the cavity bottoms. Then expose the negative mold to ultraviolet light (405 nm) for 2 min for crosslinking and curing. After thorough drying, the mold was gently peeled away to yield intact Cur@KNN MN patches. Digital photos are taken by mobile phones. Stereoscope and Gemini SEM 300 revealed the architecture of the microneedles, while EDX line- and area-scans mapped elemental distribution. For further observation, we added fluorescent dye (Alexa Fluor 488) to the MN precursor solution and observed it under a fluorescence microscope (Thunder, LEIKA, USA) after successful demolding preparation.

### Drawing of standard curve and the release of Curcumin

Weigh a certain amount of curcumin reference substance and dilute it with deionized water to prepare solutions with mass concentrations of 2, 1.5, 1, 0.5, and 0.25 µg/mL. Use a UV spectrophotometer to perform a full wavelength scan of 400–500 nm. Take the wavelength of the irradiation light corresponding to the maximum absorbance value as its maximum absorption wavelength, that is, measure its absorbance at 430 nm. Plot a standard curve with absorbance as the vertical axis and mass concentration as the horizontal axis. Then to assess the release characteristics of curcumin, MN was immersed in PBS (pH 7.4) and DMEM. The samples were then agitated at 37 °C and 100 rpm for different time periods. Subsequently, the supernatant was collected and centrifuged, and the absorbance (430 nm) was measured using a UV–vis spectrophotometer. Finally, the cumulative release of curcumin was calculated. Drug Loading Capacity (DLC, %) = mass of drug/(mass of carrier material + mass of drug) × 100%.

### Mechanical strength tests

Pressure-displacement curves were plotted utilizing a universal testing machine. Briefly, the patches were positioned on the lower platform of the machine. Subsequently, the upper platform, equipped with a transducer, was gradually moved toward the patches at a controlled rate of 1 mm/min. The force applied to the patches was recorded in real time during the test.

### Ex vivo skin insertion test

Evaluate the efficiency and penetration depth of MNs using a pigskin model. Use uniform pressure to press the MNs into the pigskin tissue, remove MNs, and apply ink on the surface of the tissue. Then take photos and record the distribution of puncture points. The penetration efficiency is obtained by dividing the number of micropores on the pigskin by the number of MN tips. Subsequently, the treated skin was dehydrated with a 30% sucrose solution, absorbing surface moisture, and embedded with OCT. Prepare tissue sections using a frozen sectioning mechanism and observe the morphology and penetration depth of microchannels under an optical microscope.

### In vitro microneedle degradation test and adhesion test

Microneedle degradation test: Attach MNs to the surface of chicken, remove them at different time points, and compare the morphological changes before and after implantation using a stereomicroscope.

Adhesion test: Following microneedle insertion, ex vivo tissues were subjected to manipulation including lifting, bending, twisting, and stretching to evaluate the attachment stability of the microneedle patches.

### Piezoelectric performance evaluation

In this study, we characterized the piezoelectric properties of Cur@KNN MN by d33 piezoelectric coefficient, ferroelectric hysteresis loop, and piezo response force microscopy. To evaluate its ferroelectric hysteresis loop, the sample was first bonded to a highly conductive adhesive tape and then polarized at a voltage of 10 kV mm⁻¹ for 30 min at 25 °C. Subsequently, the ferroelectric hysteresis loop was recorded using a PPM20816-860 analyzer.

### Piezoelectric voltage response

Cur@KNN hydrogel was cast into 1 × 1 cm square sheets. To evaluate its piezoelectric properties, two 2 × 1 cm aluminum foils with a thickness of 0.1 mm were cut and used as microelectrodes, which were clamped on both sides of the hydrogel to collect electrical signals. Fix the ultrasound probe 2 cm directly above the patch and connect the test clips of the high-precision oscilloscope (DS091304A, Agilent, USA) to the aluminum foil electrodes on both sides. Ultrasonic stimulation was initiated, and electrical signals were simultaneously collected; each frequency parameter was measured three times, with a recording duration of 5 s for each time, and the data after signal stabilization were subjected to statistical analysis. Based on the output voltage at different frequencies, an “ultrasonic frequency-electrical output” characteristic curve was plotted.

### CCK-8 assay

RAW 264.7 cells were seeded into a 96-well plate at a density of 1 × 10^4^ cells per well and cultured in DMEM containing 20 µM of curcumin and 40 µM of MN. Cells without PBS treatment served as controls. After 24 h of incubation, CCK-8 reagent was added. The light absorption value of the liquid at 450 nm was measured using a microplate reader (Bio-Tech).

### Cell death staining

The co-cultured RAW264.7 macrophages were washed and stained with a fluorescent dye mixture (Calcein-AM/PI, 1:1). The dead and live cells was then observed under a fluorescence microscope (Thunder, LEIKA, USA).

### ROS detection

Cells (1 × 10⁵/well) were cultured overnight on small disks in 24-well plates, stressed with 2 mM H_2_O_2_. After intervention stimulation, then loaded with 10 µM DCFH-DA for 20 min. ROS were visualized by fluorescence microscopy and quantified by flow cytometry.

### Angiogenesis experiment

The vascular chip was purchased from Shenzhen Xirui Biotechnology Co., Ltd. Fibrinogen (Cat# F8630-5G, Sigma-Aldrich) and thrombin (Cat# MB1368-1, Meilunbio) were procured commercially. the microfluidic-chip seeding protocol is omitted here. Next, DMEM with curcumin (20 µM) or MN (40 µM) was added. After a period of time, the cells were examined under a light microscope, and the efficiency of tube formation was calculated. Finally, the co-cultured cells were stained with a fluorescent dye (F-actin and DAPI) and photographed by a fluorescence microscope (Thunder, LEIKA, USA).

### Scratch experiment

Cells were seeded into a 24-well plate at a density of 1 × 10^5^ cells per well and cultured to confluence. The cell monolayer was scratched across the center with a 200 µL pipette tip. The cells were then incubated in a different conditioned medium. After 24 h, the migration behavior was observed under a light microscope. ImageJ was employed to measure the scratch area and calculate the ratio of the scratch area at 24 h to that at 0 h.

### Establishment of a rat model of MOUI

To establish a rat model of MOUI, adult female Sprague-Dawley rats (6 weeks, average weight 150 g) were housed in a temperature-controlled room (20–22 °C) with a 12 h light/dark cycle. Firstly, rats were anesthetized via intraperitoneal injection of Serazine Hydrochloride (5 mg/kg) and Zoletil (15 mg/kg). All subsequent operations were performed under anesthesia. The surgical procedure involved exposing the right uterine horn. An incision approximately 0.5 cm in length was made at 1/3 point from the bifurcation of the uterus. A micro-curette with a diameter of 2.5 mm was then used to thoroughly scrape the uterine cavity, with the scraping performed about 4–6 times. Finally, the uterine incision was sutured and disinfected to complete the procedure.

### Cur@KNN MN patch intervention and effect assessment

Rats synchronized in estrus were randomized into four group: sham group, MOUI group, Cur group, and Cur@KNN MN group. In the Cur/Cur@KNN group, immediately place Cur (100 mg/kg body. wt.)/MNs (200 mg/kg body. wt.) in the wound area before suturing to fill the entire area. After suturing, the Cur@KNN group was given low-intensity transabdominal low-intensity ultrasound. On day 14 and 28, the rats were sacrificed, and uteri were assessed by H&E, Masson, and immunofluorescence staining of α-SMA, MVD and VEGF. After 28 days, mate with sexually mature male rats in a 2:1 ratio. After 10 days of pregnancy, evaluate the position of embryos in each group. The ultrasonic device used in this group is US PRO2000^™^ (Model # DU3035). The device has a temperature protection function, and when the temperature exceeds 42 ℃, the instrument will stop working. In this study, the frequency used was 1.0 MHz, the intensity was 0.80 W/cm², the duty cycle was 50%, and the duration was 5–10 min.

### Statistical analysis

The data in this work came from replicated independent experiments and were analyzed using GraphPad Prism 8 software. One-way ANOVA was used to determine statistical significance. Data analysis was considered statistically significant when *P* < 0.05.

## Electronic Supplementary Material

Below is the link to the electronic supplementary material.

Supplementary Material 1

## Data Availability

Data will be made available on request.
