# Supplementary material for: Piezo-herbal microneedle patches enable wireless endometrial regeneration and fertility recovery
Source: J Nanobiotechnology. 2026 Mar 26;24:416. doi: 10.1186/s12951-026-04313-5 (PMC13141547; doi:10.1186/s12951-026-04313-5)
Supplement: Supplementary file 1 — Supplementary Material 1 [file 12951_2026_4313_MOESM1_ESM.docx]

**
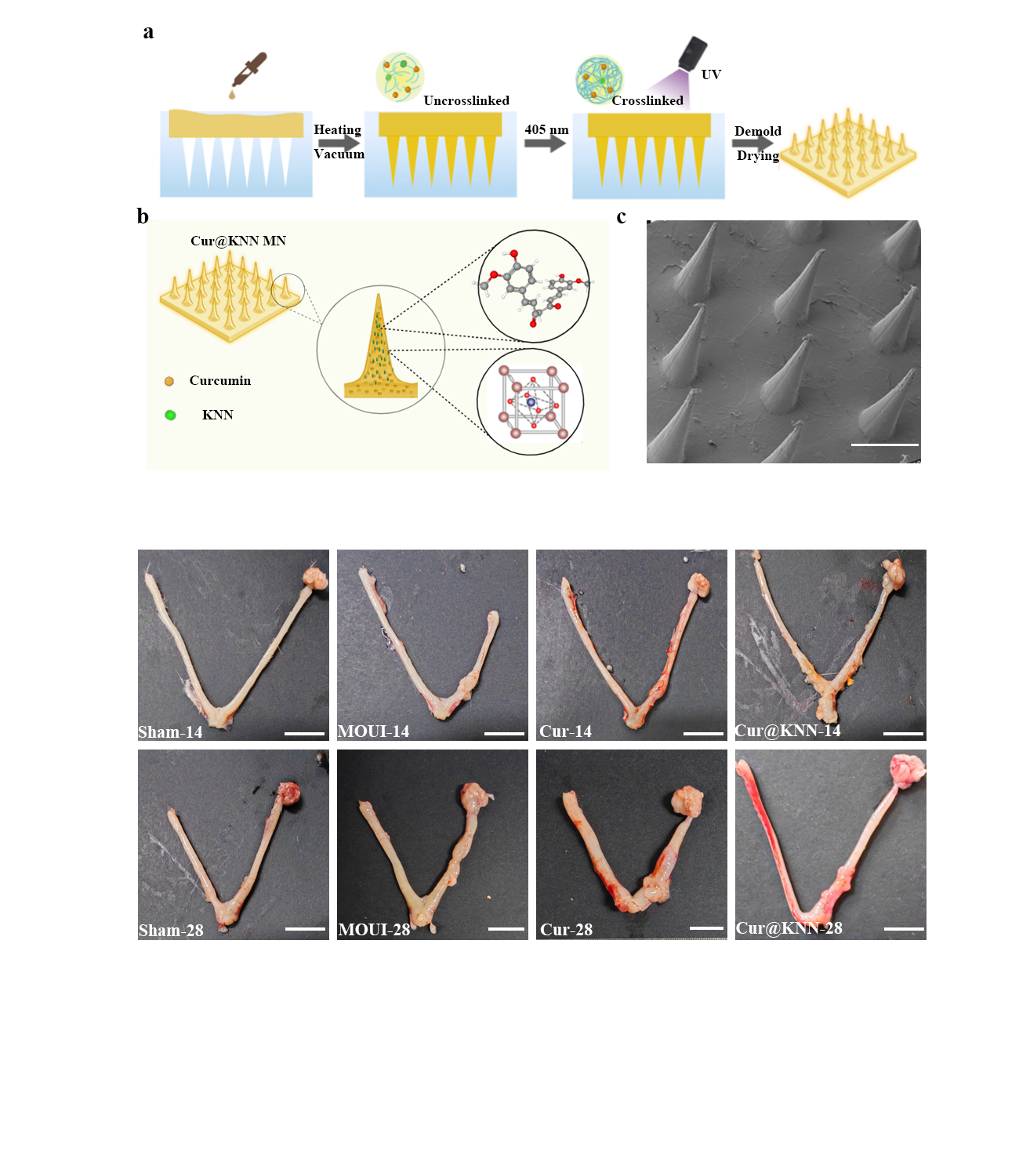
Figures**

**Figure S1. The characteristic of MNs.** a, Detailed preparation process of Cur@KNN MN patch system. b, Schematic representation of designed MN loaded with curcumin and KNN. c, The SEM of MNs. Scale bar, 500 μm.


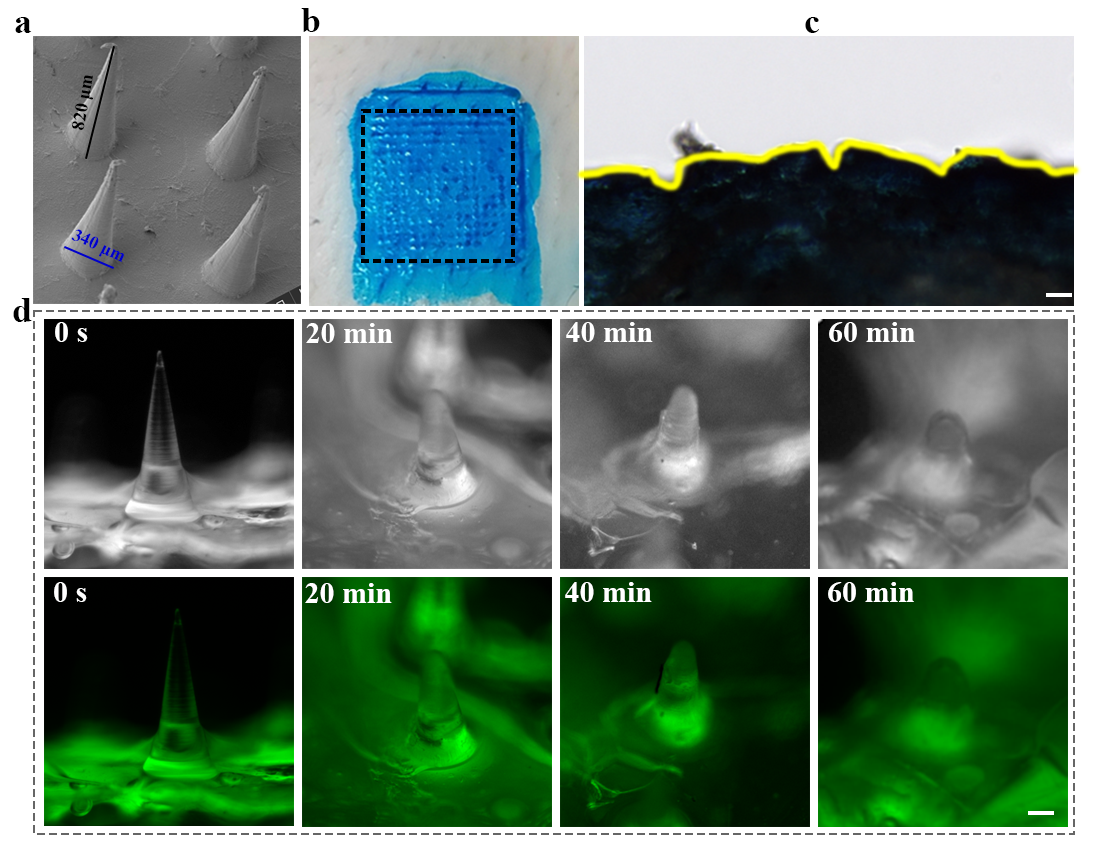


**Figure S2. The penetration and degradation of MNs.** a, The SEM and size of MNs. b, Optical image of the corresponding micropores left after removing MNs. c, Frozen section of pig skin shows MNs penetration depth. The yellow curve outlines the contour of the skin surface. Scale bar, 200 μm. d, The degradation timeline of the needles. Scale bar, 200 μm.


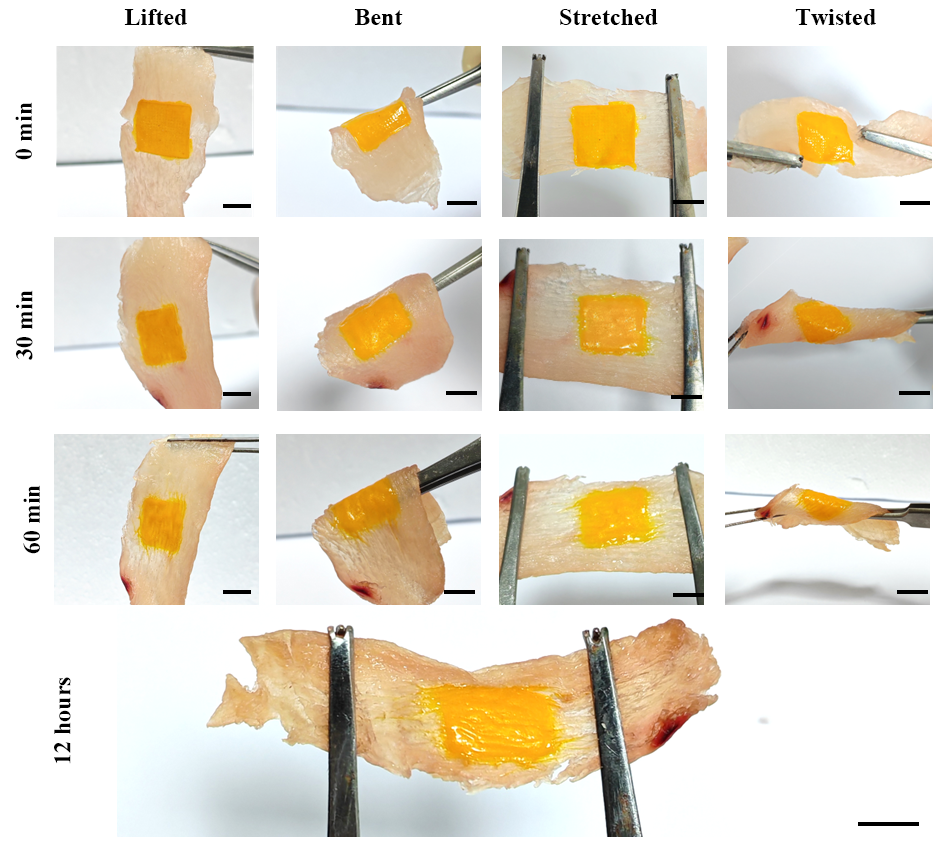


**Figure S3. The adhesion of MNs.** Photos of arrowhead MNs sticking to the chicken breast when the chicken breast is lifted, bent, twisted, or stretched.


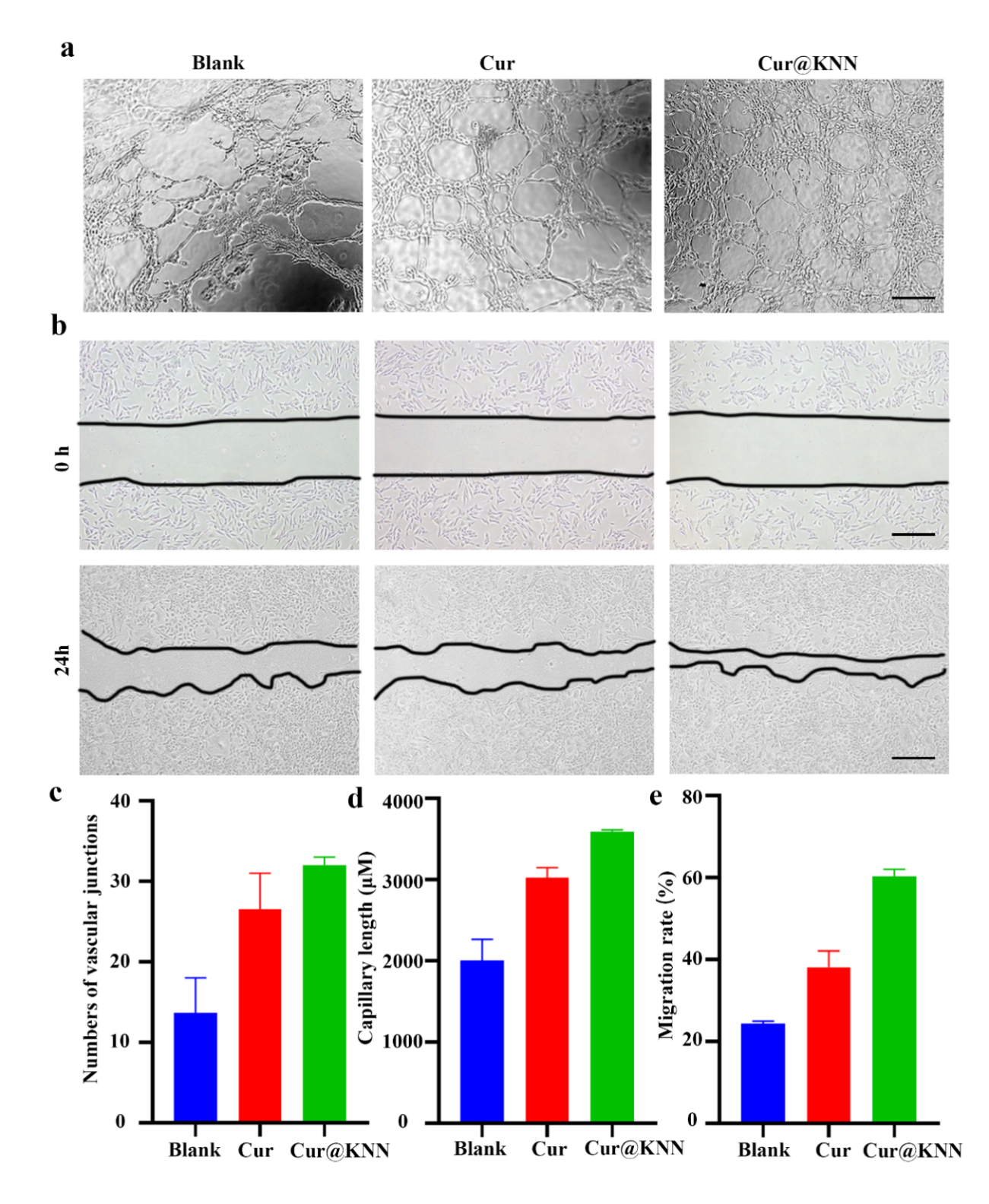
**Figure S4. The biological functions of MNs.** a, Representative images of tube formation assay showing. Scale bar, 200 μm. b, Representative images of the wound-healing assay at different time points. Scale bars, 400 μm. c-d, Quantification of number of vascular junctions and capillary length using ImageJ angiogenesis analyzer plug-in. e, Quantitation of the wound healing assay results.


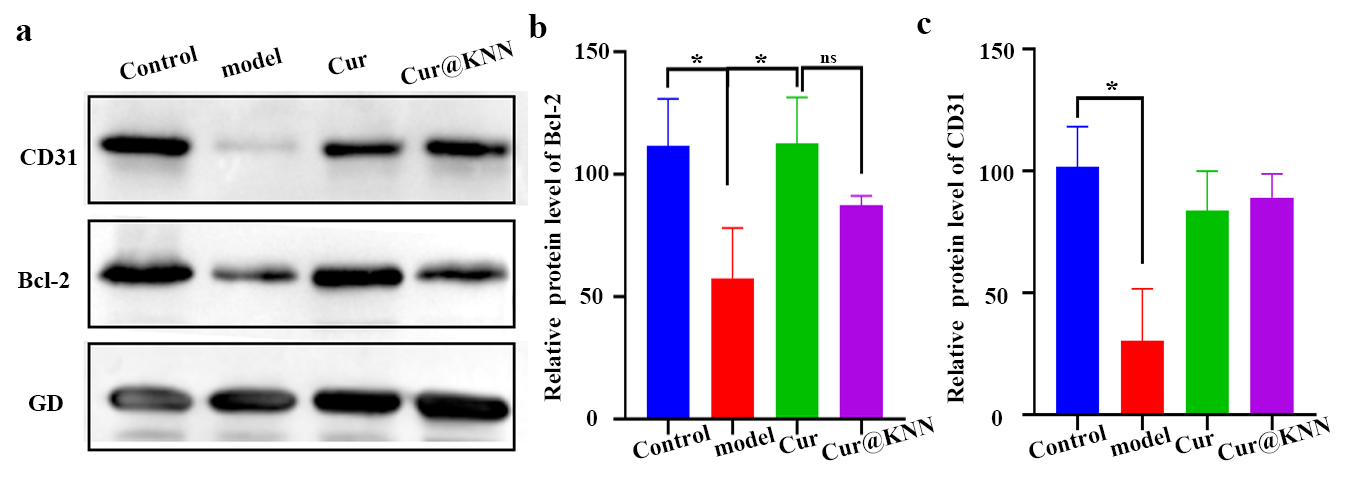
 **Figure S5. Western blot of CD31 and Bcl-2 protein expression.** a, Western Blot of the protein expressions (CD31 and Bcl-2). b-c, Quantitative analysis of protein expression. **P* < 0.05.


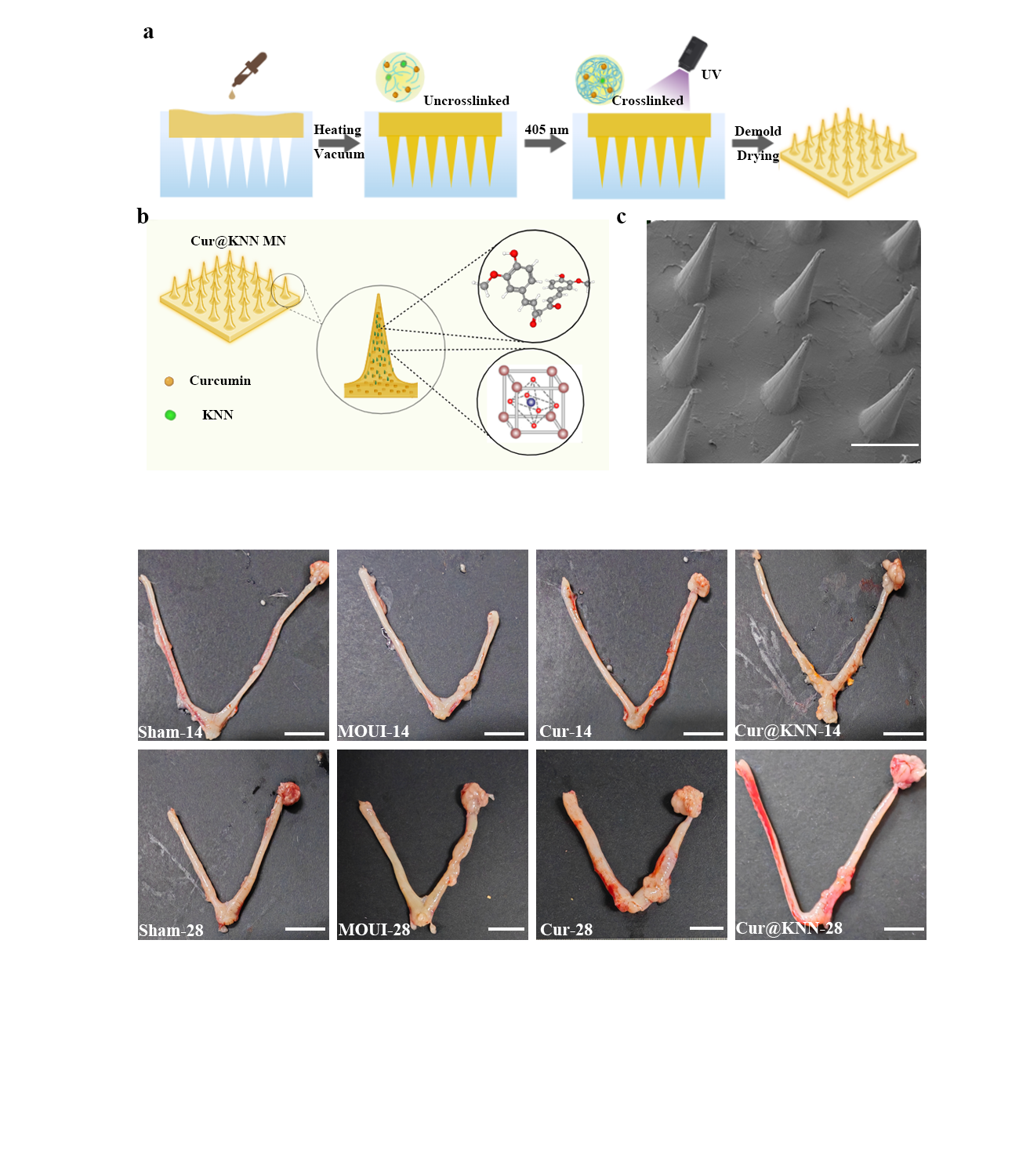


**Figure S6.** Gross image of the uterus with different treatments.


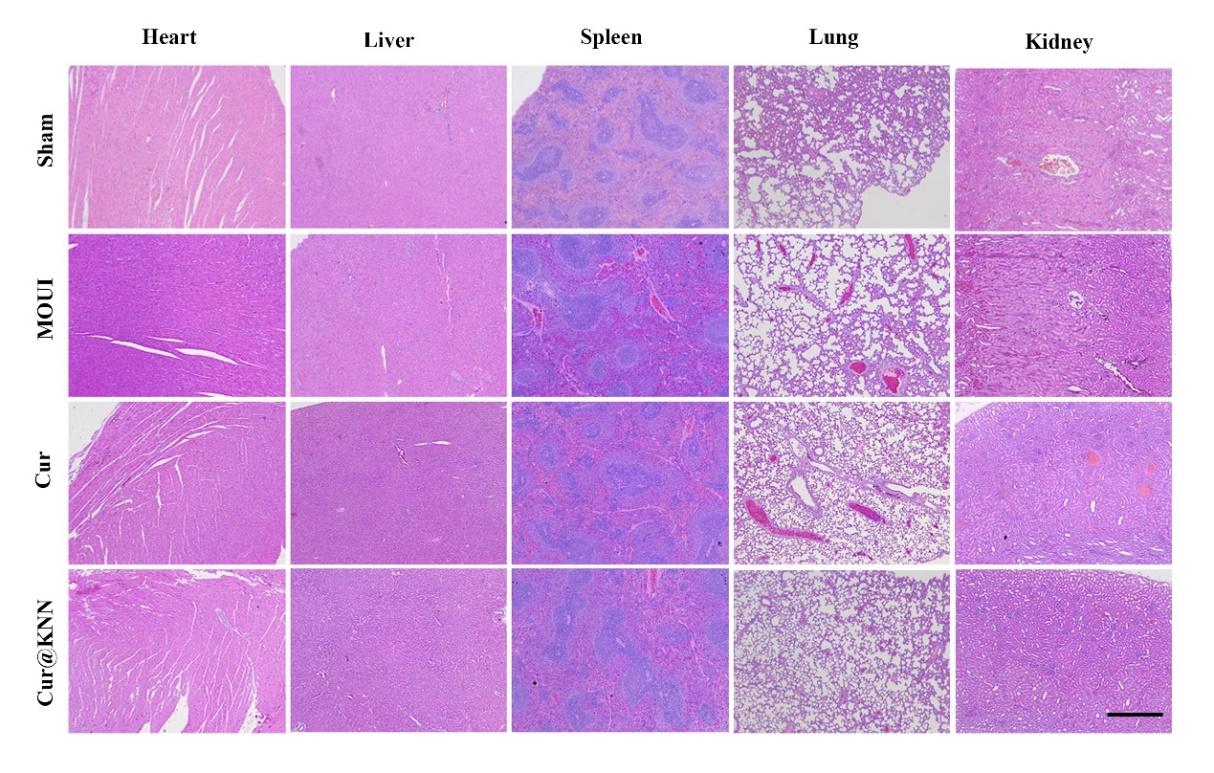
**Figure S7.** H&E staining images of the heart, liver, spleen, lung, and kidney tissue in different groups. Scale bar, 100 μm.
